# Hepatic Small Vessel Neoplasm: A Case Report and Review of the Literature

**DOI:** 10.7759/cureus.41563

**Published:** 2023-07-08

**Authors:** Adam Mylonakis, Panagiotis Sakarellos, Eleandros Kyros, Nikolaos Kydonakis, Emmanouil Mylonakis, Lysandros Karydakis, Alexandros Papalampros, Evaggelos Felekouras

**Affiliations:** 1 Department of Surgery, Laikon General Hospital, National and Kapodistrian University of Athens, Athens, GRC; 2 Department of Surgey, Laikon General Hospital, National and Kapodistrian University of Athens, Athens, GRC; 3 Department of Surgery, Laikon General Hospital, National and Kapodistrian University of Athens, Athenes, GRC

**Keywords:** differential diagnostic process, vascular neoplasm, hepatic neoplasm, hepatic small vessel neoplasm, liver

## Abstract

Hepatic small vessel neoplasm (HSVN) is a recently described vascular neoplasm of the liver. It demonstrates an infiltrative growth pattern and lacks cytologic atypia and mitotic activity. So far, no cases of metastasis or disease recurrence after excision have been reported in the literature. In this report, we present the case of a 31-year-old woman with a lesion in segments VII-VIII of the liver who was referred to our surgical department due to right lumbar pain. She underwent an atypical wedge hepatectomy (segments VII, VIII) and cholecystectomy. The histopathology of the resected specimen confirmed a 40mm HSVN. The patient did not receive any adjuvant therapy and is scheduled for follow-up with serial magnetic resonance imaging (MRI) scans over the next five years due to the unknown malignant potential of the tumor.

## Introduction

Hepatic vascular tumors cover a wide spectrum of benign and malignant lesions ranging from the common cavernous hemangioma to the rare and aggressive hepatic angiosarcoma. This category of liver tumors also includes Kaposi's sarcoma, hemangiopericytoma, epithelioid hemangioendothelioma, and the recently described hepatic small vessel neoplasm (HSVN) [1].

HSVN is a low-grade vascular neoplasm of undetermined malignant potential, first described by Gill et al. in 2016 [2]. It is a tumor composed of tightly packed vascular channels demonstrating an infiltrative growth pattern, while it lacks cytologic atypia and mitotic activity. Current data are limited; however, HSVN appears to have a benign clinical course with no reported evidence of recurrence after resection [3]. In the present work, we report the case of a 31-year-old woman referred to our surgical department due to an HSVN, and we review our considerations on this rare tumor. 

## Case presentation

**Figure 1:**
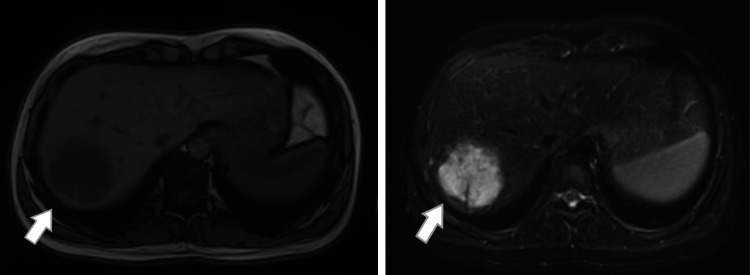
Axial T1 FL2d ΜRI image of the liver and axial T2 fat suppressed MRI image of the liver; white arrow shows a segment VII lesion

 A 31-year-old woman was referred to our department with a six-months history of pain in the right lumbar area. The patient's medical history and physical examination of the chest and abdomen were unremarkable. Chest X-ray also came back normal. Hematological and liver function tests were within normal ranges. A contrast-enhanced computed tomography (CT) scan showed an inhomogeneous lesion of approximately 5.7x4.7cm in segments VII-VIII of the right lobe of the liver. The lesion exhibited strong contrast enhancement in the arterial phase, remained strongly hyperdense in the portal venous phase, and became isodense in the late acquisitions (pooling). It was surrounded by a halo and showed multiple arterial feeding vessels. In the MRI series, the lesion exhibited a low signal on T1 sequences and a high signal, along with the presence of a central area with a low signal on T2 sequences (Figure 1). On dynamic testing, the lesion showed remarkable inhomogeneous contrast enhancement during the arterial phase, with accompanying areas of transient peripheral perfusion disturbance and progressive depletion, which was evident in the late phase.

**Figure 2:**
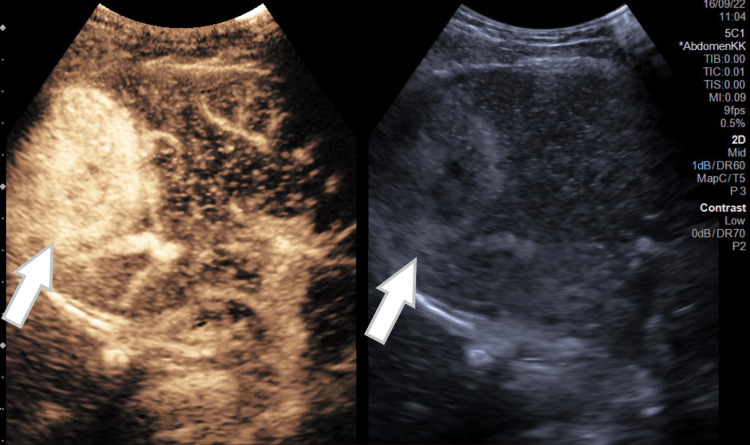
Contrast-enhanced ultrasound (CEUS) of the liver; white arrow shows a segment VII lesion

Abdominal ultrasound demonstrated a solid hypoechoic lesion with a smooth border, with peripheral and central vasculature. After intravenous administration of contrast agent (Sonovue, Bracco International BV, Milan, Italy), contrast-enhanced ultrasound (CEUS) showed in the arterial phase (13 sec) a strong uptake from the periphery to the center with complete uptake at 20 sec (Figure 2). The lesion remained hyperechoic compared to the liver parenchyma in the portal and prolonged portal phases.

**Figure 3:**
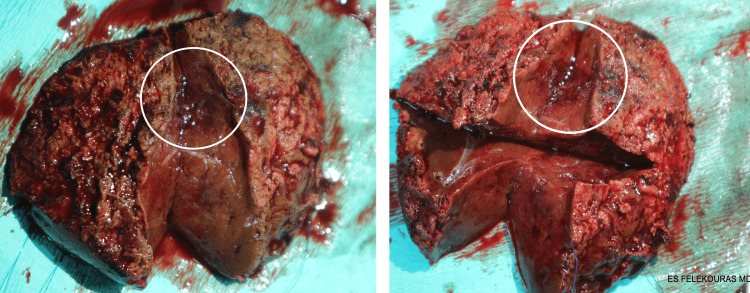
Gross photograph of surgical specimen of HSVN (circle) demonstrating a vascular unencapsulated tumor with a poorly circumscribed border

The patient underwent an atypical wedge hepatectomy (segments VII, VIII) (Figure 3) and cholecystectomy through a Chevron incision. Intraoperatively the lesion was identified using intraoperative ultrasound. Notably its size significantly decreased after disruption of its vascular supply. Postoperatively, the condition of the patient was complicated with fever and an intra-abdominal abscess in the bed of the hepatectomy was revealed on CT scan. The patient underwent an ultrasound guided percutaneous drainage of the abscess and was discharged on the 14th postoperative day.

**Figure 4:**
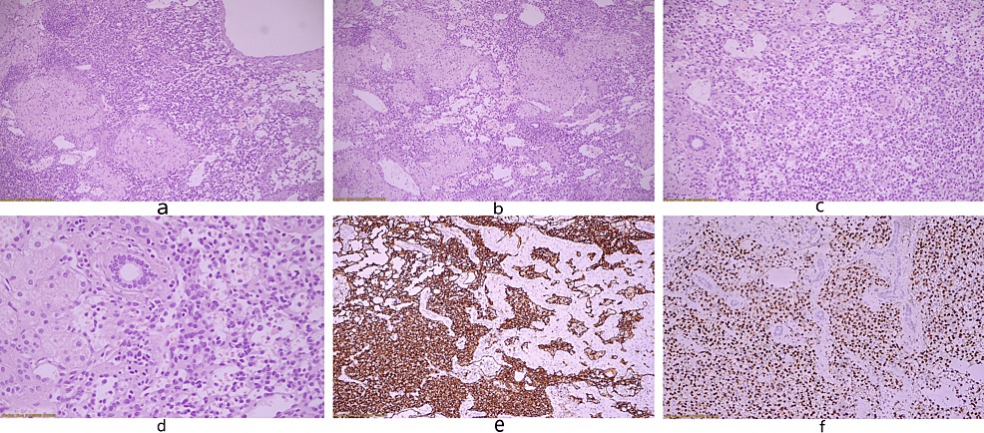
Microscopic appearance of the neoplasm H&E stain x100 (a, b), x200 (c), x400 (d), erythroblast transformation specific related gene (ERG) stain x200 (e), CD31 stain x100 (f); Proliferation of small, thin-wall vessels infiltrating hepatic parenchyma (a, b). Portal tracts are entrapped within the neoplasm (c). Higher magnification reveals plump, ovoid endothelial cells with no significant nuclear atypia lining neoplastic vessels (d). Neoplastic cells stain positive for ERG (e) and CD31 (f), confirming the vascular nature of the neoplasm.

The resected specimen was a vascular neoplasm measuring 4cm at its maximum diameter, mostly consisting of small-diameter, thin-walled vascular spaces lined by oval cells of endothelial origin. The tumor developed infiltratively between the hepatic trabeculae and enclosed portal spaces and interlobular cholangia, extending at a great distance from the main tumor, focally reaching its surgical border. Papillary formation, layering, nuclear pleomorphism, mitotic activity, or necrosis of neoplastic cells were not observed (Figure 4).

Immunohistochemistry confirmed the vascular nature of the neoplasm (positive expression of CD31, CD34, ERG). The CAMTA1 marker for epithelioid hemangioendothelioma, as well as staining for c-Myc and p53, were negative. The cell proliferation index Ki67 was difficult to calculate due to lymphocytic infiltration, but it was estimated to be less than 10%. These findings were consistent with a hepatic small vessel neoplasm. The patient is currently scheduled for an initial follow-up with CEUS and MRI six months after resection, with close surveillance planned for the next five years.

## Discussion

Hepatic small vessel neoplasm is a vascular tumor of the liver that has been recently recognized. However, its histopathology and clinical behavior have not been adequately established in the medical literature. Its true incidence and long-term prognosis remain unclear, and case reports and series present diverse histopathological descriptions, utilize different imaging modalities, and focus on varied aspects of this pathological entity. To our knowledge, there have been 25 cases (Table 1) detected so far since the first case series in 2016 reported by Gill et al. [2]. Goh et al., in their meta-analysis, demonstrated a male predominance with a male-to-female ratio of 2,8:1 with a median age of 58 years (range 24-83). The majority of cases involved solitary lesions; five patients exhibited multiple lesions [2,4,5], while Cicala et al. [5] reported possible disease dissemination to the spleen. The size of the tumors ranged from 10 mm to 159 mm, with a median size of 27.8 mm at its greatest diameter. Our case involves a 31-year-old female with a six-month history of lumbar pain presenting with an HSVN of segments VII and VIII.

**Table 1:** Hepatic Small Vessel Neoplasm patient characteristics M - male; F - female; HCV - hepatitis C virus; HCC - hepatocellular carcinoma; NAFLD - non-alcoholic fatty liver disease; CHF - congestive heart failure; FNH - focal nodular hyperplasia; RFA - radiofrequency ablation; TACE - transcatheter arterial chemoembolization; N/D - not described

Author	Sex	Age	Size (mm)	Clinical Presentation	Intervention
Gill et al. [2]	M	83	15	Incidental finding	None
Gill et al. [2]	M	58	7	Incidental finding on an HCV patient	Resection
Gill et al. [2]	M	57	20	Incidental finding	N/D
Gill et al. [2]	M	47	11	Incidental finding on a patient with renal cell carcinoma	Resection
Gill et al. [2]	M	53	2	Multiple hypervascular liver tumors on imaging	None
Gill et al. [2]	F	58	15	Three incidental liver tumors at autopsy	None
Gill et al. [2]	F	37	55	Incidental liver tumor on a pregnant patient	Resection
Gill et al. [2]	M	61	13	Imaging suggestive of HCC on an HCV patient	None
Gill et al. [2]	M	43	22	Growing lesion on a patient with NAFLD	Resection
Gill et al. [2]	M	66	18	Imaging suggestive of HCC on an HCV and cirrhotic patient	Hepatectomy
Gill et al. [2]	M	65	22	Imaging of possible metastatic tumor on a patient with bronchial carcinoid	Wedge biopsy +RFA
Gill et al. [2]	M	54	42	Incidental finding	TACE followed by resection
Gill et al. [2]	F	59	25	Incidental finding on a patient with CHF and renal failure	Resection
Gill et al. [2]	F	24	10	Resection of 5.3 cm hepatocellular adenoma on a patient with NAFLD	N/D
Gill et al. [2]	M	67	27	Elevated LFT and imaging suggestive of FNH	None
Gill et al. [2]	M	77	30	Incidental finding	None
Gill et al. [2]	M	65	2	Incidental finding on a cirrhotic patient	Hepatectomy
Mullholand et al. [4]	M	57	27	Incidental finding	Laparoscopic posterior hepatectomy (segments VI and VII) and cholecystectomy
Cicala et al. [5]	F	70	20	Incidental finding of multiple hypodense hepatic lesions	Biopsy, Wait-and-see
Rangaswamy et al. [6]	F	48	N/D	Incidental finding	N/D
Rangaswamy et al. [6]	F	67	N/D	Incidental finding	N/D
Koschny et al. [7]	M	37	2.6	Fatigue, pruritus, history of Crohn's disease, and liver hemangiomas	Atypical segmental hepatectomy
Walcott- Sapp et al. [8]	M	62	159	Epigastric fullness	Resection
Lewis et al. [9]	F	59	22	Epigastric abdominal pain on a cirrhotic patient	Biopsy
Present case	F	31	40	Lumbar pain	Atypical wedge hepatectomy (segments VII and VIII) and cholecystectomy

Although HSVN exact etiology is still vague, the activating hotspot of GNAQ mutation seems to be an important step in HSVN tumorigenesis and malignant potential [2]. The role of this mutation in endothelial cell biology is under research, but it appears to cause capillary malformation, as it is exhibited in Sturge-Weber syndrome [10].

Clinical presentation of patients with HSVN is often occult, with the majority of the lesions detected as incidental findings. Patients may complain of fullness, abdominal pain, or fatigue or exhibit abnormal liver function tests. Our patient was referred to our surgical department with a history of right lumbar pain lasting for six months.

On radiological imaging, HSVN displays an early peripheral continuous enhancement with persistent enhancement in portal venous and late phases [6]. These findings can help differentiate HSVN from hemangioma, as the latter displays a peripheral globular discontinuous enhancement [5]. On MRI imaging, HSVN exhibits a high and homogeneous signal in T2 weighted sequences, helping in the differential diagnosis of a hepatic angiosarcoma that exhibits a high but heterogeneous signal in T2 sequences [11].

As far as morphologic features are concerned, hepatic small vessel neoplasm is an infiltrative tumor composed of thin-walled small vascular spaces lined by flat to plump-oval endothelial cells. The cells comprising the lesion lack features of cytologic atypia, such as mitotic activity, necrosis, or nuclear irregularity/pleomorphism [2]. Immunohistochemistry features of HSVN include positive vascular markers (CD43, CD31, and FLI-1) [2], with weak or negative c-Myc and p53 expression [3]. In their study, Gill et al. reported the use of the Ki-67 proliferative index as a discriminator between angiosarcoma and HSVN. Setting a cutoff level of 10%, they reported a 100% sensitivity and specificity for distinguishing these two entities [2].

In our case, the diagnostic workup included blood and liver function tests, a CT scan and MRI of the abdomen, as well as contrast-enhanced ultrasound, which raised the suspicion of HSVN. We performed an atypical wedge hepatectomy (segments VII, VIII) and a cholecystectomy under intraoperative ultrasound guidance.

We noticed that, after the disruption of its vascular supply, the tumor decreased in size, making it difficult to distinguish from normal liver parenchyma. Thus, we highlight the importance of preoperative imaging and surgical planning, as well as the use of intraoperative ultrasound to locate the lesion and ensure negative resection margins.

Furthermore, we propose that all cases of suspected or diagnosed HSVN be referred to tertiary hospitals with a specialized hepato-pancreato-biliary (HPB) unit. We underline the importance of a multidisciplinary team (MDT) approach involving an oncologist, surgeon, diagnostic and interventional radiologist, hepatologist, pathologist, endoscopist, and gastroenterologist, given the rarity of these newly described tumors.

At present, more data and case series are required to establish safe diagnostic criteria and therapeutic guidelines for hepatic small vessel neoplasms. Therefore, we suggest tumor resection followed by close and long-term follow-up for these patients.

## Conclusions

HSVN, or hepatic small vessel neoplasm, is a vascular neoplasm of the liver characterized by an infiltrative growth pattern. Based on the currently available data, it appears to have a benign clinical course. More research and case series are essential for establishing reliable diagnostic criteria as well as therapeutic and follow-up guidelines.
